# MUC1 Mucin: A Putative Regulatory (Checkpoint) Molecule of T Cells

**DOI:** 10.3389/fimmu.2018.02391

**Published:** 2018-10-22

**Authors:** Babita Agrawal, Nancy Gupta, Jeffrey D. Konowalchuk

**Affiliations:** Department of Surgery, Faculty of Medicine and Dentistry, University of Alberta, Edmonton, AB, Canada

**Keywords:** T cells, MUC1 mucin, immune regulation, checkpoint molecule, coinhibitory molecule

## Abstract

T lymphocytes are at the center of inducing an effective adaptive immune response and maintaining homeostasis. T cell responses are initiated through interactions between antigen presenting cells (APCs) and T cells. The type and strength of signals delivered through the T cell receptor (TCR) may modulate how the cells respond. The TCR-MHC (T cell receptor-major histocompatibility complex molecules) complex dictates the specificity, whereas co-stimulatory signals induced by interaction of various accessory cell surface molecules strengthen and optimize T cell responses. Multiple immune regulatory mechanisms brought about by co-inhibitory molecules expressed on T cells play a key role in orchestrating successful and non-damaging immunity. These co-inhibitory molecules are also referred to as initiators of immune check-points or co-inhibitory pathways. Knowledge of co-inhibitory pathways associated with activated T lymphocytes has allowed a better understanding of (a) the inflammatory and anti-inflammatory processes associated with infectious diseases and autoimmune diseases, and (b) mechanisms by which tumors evade immune attack. Many of these regulatory pathways are non-redundant and function in a highly concerted manner. Targeting them has provided effective approaches in treating cancer and autoimmune diseases. For this reason, it is valuable to identify any co-inhibitory molecules that affect these pathways. MUC1 mucin (CD227) has long been known to be expressed by epithelial cells and overexpressed by a multitude of adenocarcinomas. As long ago as 1998 we made a surprising discovery that MUC1 is also expressed by activated human T cells and we provided the first evidence of the role of MUC1 as a novel T cell regulator. Subsequent studies from different laboratories, as well as ours, supported an immuno-regulatory role of MUC1 in infections, inflammation, and autoimmunity that corroborated our original findings establishing MUC1 as a novel T cell regulatory molecule. In this article, we will discuss the experimental evidence supporting MUC1 as a putative regulatory molecule or a “checkpoint molecule” of T cells with implications as a novel biomarker and therapeutic target in chronic diseases such as autoimmunity, inflammation and cancer, and possibly infections.

## MUC1 mucin molecule as a cancer-associated antigen

**Figure 1:**
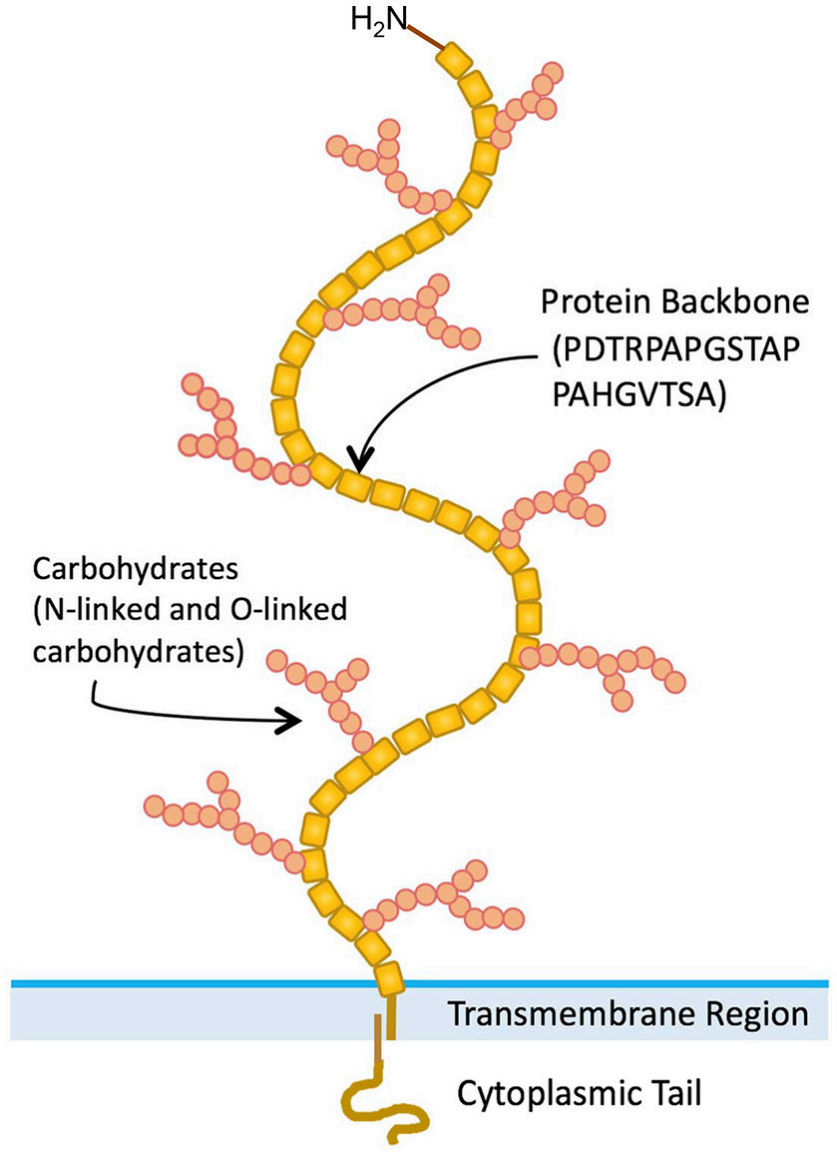
Structure of MUC1 mucin.

A variety of normal and malignant epithelial cells express mucins, which are large (>200 kDa) glycoproteins with a high carbohydrate content (50–90% by weight) (1). Twenty-two mucin genes have been cloned [(2, 3) HUGO Gene Nomenclature Committee, https://www.genenames.org/cgi-bin/genefamilies/set/648]. A large number of tandem repeat (VNTR) amino acid sequences are present in variable numbers in the extracellular domains of mucin proteins (4). Presence of numerous serine and threonine residues in mucin VNTR sequences, provide potential O-glycosylation sites. The MUC1 mucin (CD227) gene has been cloned and extensively characterized (5). MUC1 is a polymorphic mucin-like protein that contains a large extracellular domain consisting of a glycosylated polypeptide made up of 30–100 tandem repeats of a 20-amino acid sequence, a transmembrane domain, and a cytoplasmic tail (Figure 1) (6). After translation, MUC1 protein is modified extensively by O-linked sugar moieties. Also after translation, proteolytic cleavage produces two products (7). These form heterodimer complexes that are composed of a large extracellular domain linked by non-covalent, SDS-sensitive bonds to the smaller protein molecule, which includes the transmembrane and cytoplasmic domains (7).

The MUC1 protein is involved in cell-cell and cell-extracellular matrix interactions, and may play a role in the metastatic spread of cancer cells from the initial tumor site (8). It has been demonstrated that MUC1 is involved in oncogenic processes and enhances tumor initiation and progression (9). Mucin-like molecules have been associated not only with passively mediating adhesion and migration, but also with an active signaling function (10). Endothelium mucin-like molecules e.g., CD34, GlyCAM1, PSGL1, and MadCAM1 (11) have been shown to be involved with lymphocyte trafficking and transfer of signal to the cell nucleus upon interaction with their ligands. Many other additional functions have been proposed for MUC1. MUC1 has also been suggested to function in epithelial morphogenesis and tumor progression due to its extensive expression in secretory epithelial tissues from mid-gestation throughout adulthood and elevated level of expression found in carcinomas and metastatic lesions (12). It has been suggested that MUC1 could also function to provide steric hindrance by the large glycosylated extracellular domain (13), remodel the cytoskeletal network (14), or down-regulate signaling events via activities of catenins, cadherins, or integrins (15). The cytoplasmic tail of MUC1 is phosphorylated in breast cancer cells, which supports a transmembrane signal transduction function for MUC1 (16). A unique differential splicing product of MUC1, MUC1/Y, has also been identified. It has been suggested that secreted MUC1 is a ligand for MUC1/Y, which initiates signaling events and changes in cell morphology in tumor cells (7).

MUC1 mucin is normally expressed by secretory epithelial cells but has been implicated as a tumor antigen for various vaccine immunotherapeutic studies targeting the generation of B and T cell responses because it is abundantly expressed in aberrant forms by a number of adenocarcinoma tumor cells (8). MUC1-derived peptides have been suggested to be targets of MHC-restricted cytotoxic T lymphocytes (CTLs) capable of killing tumors (17). In earlier studies, MUC1 was shown to be targeted by non-classical MHC-unrestricted CTLs, based on their generation upon sequential stimulation by MHC-unmatched MUC1^+^ tumor cell lines and blocking of tumor cell killing by anti-MUC1 antibody (18). Although the accuracy of the observation is not in question, there may be an alternative interpretation of the mechanism (than previously put forward) based on the findings we discuss in this article. Several clinical trials have been done and are currently ongoing examining the potential of various formulations of MUC1-derived products, as new candidates for immunotherapy of several adenocarcinomas (19).

## T cell activation: a balance between positive and negative regulation

T lymphocytes are at the center of inducing an effective adaptive immune response and maintaining homeostasis. Interactions between antigen presenting cells (APCs) and T cells initiate T cell responses (20).

The type and strength of signals delivered through the T cell receptor (TCR) may modulate how the cells respond. The TCR-MHC (T cell receptor-major histocompatibility complex molecules) complex dictate the specificity of interaction, whereas co-stimulatory signals induced by interaction of various accessory cell surface molecules (including CD2/LFA-3, LFA-1/ICAM-1, CD28/B7, CD4/MHCII, CD8/MHCI, etc.) dictate the strength and quality of the induced responses (21). Multiple sequential steps are involved in inducing T cell immunity, such as the clonal selection of specific TCR-bearing T cells, activation and proliferation of antigen-specific T cells in secondary lymphoid tissues, and subsequent trafficking of T cells to sites of antigen exposure or insult. At the site of antigen exposure or injury, T cells execute direct effector functions and provide help to a number of effector immune cells. Each of these steps is regulated by multiple receptors, signals, and soluble factors that fine-tune the T cell response (22).

TCR engagement not only results in stimulation of T cells, its ligation also activates proteins (e.g., phosphatases), which serve as negative regulators and terminate the activation cascade initiated by phosphotyrosine kinases (23). TCR-mediated second messenger pathways also play a crucial role in maintaining homeostasis by eliminating activated T cells through apoptosis i.e., activation induced cell death (AICD) (24). Signaling through two well-known negative regulators or checkpoints of T cells, cytotoxic T-lymphocyte-associated protein 4 (CTLA-4) and programmed cell death 1 (PD-1), leads to direct inhibition of T cell responses. CTLA-4 and PD-1 bind to the B7 family of molecules B7-1 and B7-2, or PD-L, respectively, and mediate the maintenance of T cell homeostasis and peripheral tolerance. CTLA-4 dampens T cell responses at the stage of initial activation, whereas PD-1 attenuates the immune response of effector T cells, thereby minimizing self-tissue damage during immune activation (25). A number of other T cell inhibitory/checkpoint molecules have also been identified such as BTLA (B and T lymphocyte associated), KIR (Killer Immunoglobulin-like receptor), LAG-3 (lymphocyte activation gene 3), and TIM-3 (T cell membrane protein 3), which function to limit the ongoing immune responses (21).

Immune checkpoints are crucial to maintain self-tolerance (to prevent autoimmunity) and protect self-tissue from damage during an ongoing immune response required to defend against a pathogenic infection (26). During a phase of normal activation, CD4^+^ and CD8^+^ T cells express multiple immune checkpoint molecules, and some of these molecules also serve a co-stimulatory role in T cell activation (26). Also, in healthy individuals, immune checkpoint molecules expressed on T cells are tightly linked to the differentiation and/or functional state of those cells (naïve, effector, regulatory, or memory cells) (26). Therefore, the expression of checkpoint molecules on T cells does not necessarily correspond to reduced T cell function or T cell dysfunction, but may rather be associated with activated and/or functional T cells (26).

However, in the context of viral infection or the tumor microenvironment T cells chronically exposed to antigen stimulation demonstrate accumulated expression of immune checkpoint (IC) molecules such as CTLA-4, PD-1, TIM-3, and LAG-3, and are characterized as “exhausted.” The “exhausted” T cells have reduced ability to proliferate, produce cytokines and kill target cells (27). Further, it has been shown that T cell dysfunction is exacerbated upon binding of checkpoints such as PD-1 and TIM-3 to their respective ligands, whereas antibodies blocking these interactions reverse T cell dysfunction (28). In contrast, T cells obtained from individuals with autoimmune conditions have enhanced expression of these molecules that represent an activated T cell state (29).

A variety of human diseases related to immunological disorders, including graft vs. host disease, autoimmunity, infection, and cancer emphasize the importance of co-signaling and checkpoint molecules (30). T cell stimulatory as well as inhibitory molecules have found new attraction as targets for immunotherapy in cancer and some chronic viral diseases, and also as biomarkers for chronic diseases. Antibodies blocking co-inhibitory or checkpoint molecules, known as checkpoint inhibitors, have provided successful immunotherapeutic approaches for many cancers, by working on the immune system, instead of on tumor cells, to “release the brakes” (inhibitory signals) (30). As a logical extension to these applications, combinations of checkpoint inhibitors have been tested clinically (31). Even in combination with vaccine approaches, checkpoint blocking is being tested as a new approach that enhances the activation of antitumor or antiviral responses (32). At the opposite end of the spectrum, antibodies against co-stimulatory molecules are being evaluated as biomarkers and as therapeutics to dampen abnormal T cell activation in inflammatory and autoimmune diseases (33).

Multiple checkpoints may efficiently control the development of aberrant immunity, and it is possible that the immune checkpoints investigated to date are only a fragment of the receptors and ligands that have their own unique signature and mechanisms to inhibit specific types of immune responses. Therefore, identifying new putative immune checkpoints would aid tremendously to our understanding of immunobiology, as would their translation as potential biomarkers and therapeutic targets for various chronic diseases including cancer, infectious and autoimmune/inflammatory diseases.

## MUC1 (CD227) expression on human T cells: a serendipitous discovery

Highly glycosylated forms of MUC1 are expressed and secreted by a number of normal secretory epithelial cells. In the endometrium and serum of pregnant women, various glycoforms of MUC1 are present, some of which are similar to those of cancer-associated MUC1 (34). It is possible that MUC1 produced under the influence of progesterone in the endometrium might inhibit T cell responses in the reproductive tract, thereby allowing a semi-allogeneic embryo to survive, which would otherwise be rejected (35). We speculated that MUC1 functions in modulating immune responses because of the findings that (a) soluble MUC1 inhibits the attachment of eosinophils to antibody-coated targets (36), (b) soluble MUC1 inhibits T cell proliferation (37), (c) MUC1 is expressed on human myeloma cells (38), and (d) MUC1 is also expressed on mouse granular metrial gland cells, which have a lymphocyte precursor (39).

Twenty years ago, while attempting to use human T cells as negative controls for binding of an anti-MUC1 monoclonal antibody (B27.29), we surprisingly discovered that MUC1 mucin, largely considered an epithelial antigen and marker for epithelial tumor cells, is also expressed by activated human T cells (40). Initially, our observation endured some skepticism within the research community, since the observation and its implications were highly unexpected. A MUC1 expert Dr. S.J. Gendler cautiously commented, “It is going to take decades for MUC1 to be accepted as a bona-fide immunoregulatory molecule of T cells.” True to her words, almost ~20 years later, we are writing this hypothesis and theory article instituting MUC1 as an authentic T cell regulatory/coinhibitory/checkpoint molecule.

We hypothesized that along with other possible functions, MUC1 mucin expressed on T cells has an immunoregulatory function. The evidence supporting this hypothesis is: (i) MUC1 mucin is rapidly induced in the majority of activated human T cells and is expressed on the surface, (ii) after removal of the mitogenic stimulus, expression of MUC1 is down-regulated, (iii) T cell proliferative response is inhibited upon cross-linking with anti-MUC1 mAb B27.29 (and other antibodies), (iv) MUC1 mucin is secreted in the supernatants of PHA-activated human T cells, (v) co-stimulation with IL-2 or anti-CD28 antibody reverse the inhibition of T cell proliferation mediated by cross-linking with surface MUC1; (vi) MUC1 expression on T cells is increased upon treatment with pro-inflammatory cytokines; and (vii) single Fab' fragments of anti-MUC1 antibody don't inhibit T cell responses suggesting that cross-linking of MUC1 is important in signaling (40, 41). Our original findings (40) have been reproduced by several laboratories around the world (13, 42, 43).

## Potential ligands for MUC1 expressed on T cells

MUC1 has been found to bind to a number of molecules expressed on macrophages and DCs such as DC-SIGN (CD209), mannose receptor, and macrophage galactose lectin (MGL) (44). Further, MUC1 can bind to sialoadhesin (CD169, Siglec-1), an adhesion molecule expressed by macrophages (45). Domain 1 of intercellular adhesion molecule 1 (ICAM-1), which is expressed on endothelial cells and immune cells (macrophages and lymphocytes) has also been shown to be a ligand for MUC1 (46). In addition, E-selectin, a cell adhesion molecule expressed only in endothelial cells, is also a ligand for MUC1. The binding of MUC1 to E-selectin and to ICAM-1 may play a major role in T cell migration (47). Further, upon stimulation with chemokines, MUC1 molecules were found to be concentrated on the leading edge of polarized activated T cells (48). These observations suggest a role of MUC1 in early interactions between T cells and endothelial cells to initiate extravasation and migration. It has been noted that there is aberrant overexpression of MUC1 in adult T-cell leukemia/lymphoma, which plays a role in disease progression (43).

As described earlier (Figure 1), MUC1 is a large and heavily glycosylated trans-membrane protein. Our initial discovery of MUC1 expression on activated T cells was by using B27.29 antibody which recognizes the glycopeptide epitope in the extracellular tandem repeat region of MUC1 (40). Further studies reported that MUC1 expressed by activated human T cells could be recognized by HMFG1, HMPV and MF06 mAbs which recognize core 2 based O-glycans such as Gal β1-3(GlcNAc β1-6) GalNac (48). Further, it was reported that the enzymes that synthesize core 2 based O-glycans were expressed by activated T cells (48). Interestingly, it has been shown that Galectin-3 (GAL-3) is a ligand for oncofetal Thomson-Friedenreich carbohydrate (Galβ1, 3GalNAcα-, T, or TF) antigens on MUC1 mucin, which are expressed on human colon cancer cells (49). GAL-3 contains a carbohydrate recognition domain (CRD), with the ability to bind to β-galactosides. It can therefore be envisaged that MUC1 expressed by activated T cells can potentially bind to GAL-3. GAL-3 is widely distributed throughout the body both in soluble and cell-associated form, and is highly expressed on myeloid antigen-presenting cells, fibroblasts, epithelial as well as endothelial cells in cytoplasm and membrane-associated form (50). GAL-3 has been suggested to be acting as a danger associated molecular pattern (DAMP) and as a pathogen recognition receptor (PRR). It has also been shown to participate in migration, activation, modulation of inflammatory/regulatory cytokines as well as regulating apoptosis in innate immune cells (50). It is possible that GAL-3 expressed both extracellularly and widely on the surface of myeloid, epithelial and endothelial cells serves as a ligand for MUC1 expressed on activated T cells, resulting in migration and modulation of effector function. Whether this effect would be dependent or independent of TCR engagement must be examined.

In conclusion, there are several potential ligands of MUC1 mucin expressed by T cells (Figure 4), and their role in mediating T cell regulation needs to be determined to allow delineation of a physiological pathway of MUC1 mediated regulation.

## Initial evidence supporting the role of MUC1 as an immunoregulatory molecule on T cells

In our initial studies, we primarily used B27.29 monoclonal antibody as an anti-MUC1 ligand to show the expression of MUC1 on activated T cells as well as MUC1 cross-linking-mediated inhibition of T cell proliferation (40). The non-specific effect of the Fc portion of the anti-MUC1 antibody was ruled out by the use of F(ab')_2_ fragments of B27.29 in our later studies (41). Further, we analyzed 10 different anti-MUC1 antibodies specific for peptide, glycopeptide or carbohydrate epitopes, for their capability to bind to activated human T cells and inhibit T cell proliferation. All of the tested antibodies bound to activated T cells and inhibited T cell proliferation upon surface MUC1 cross-linking to varying degrees (41). We further demonstrated that providing co-stimulation with anti-CD28 or IL-2 led to reversal of the effect of anti-MUC1 mAb cross-linking, suggesting an intracellular signal-mediated negative regulatory role of MUC1 (41).

**Figure 2:**
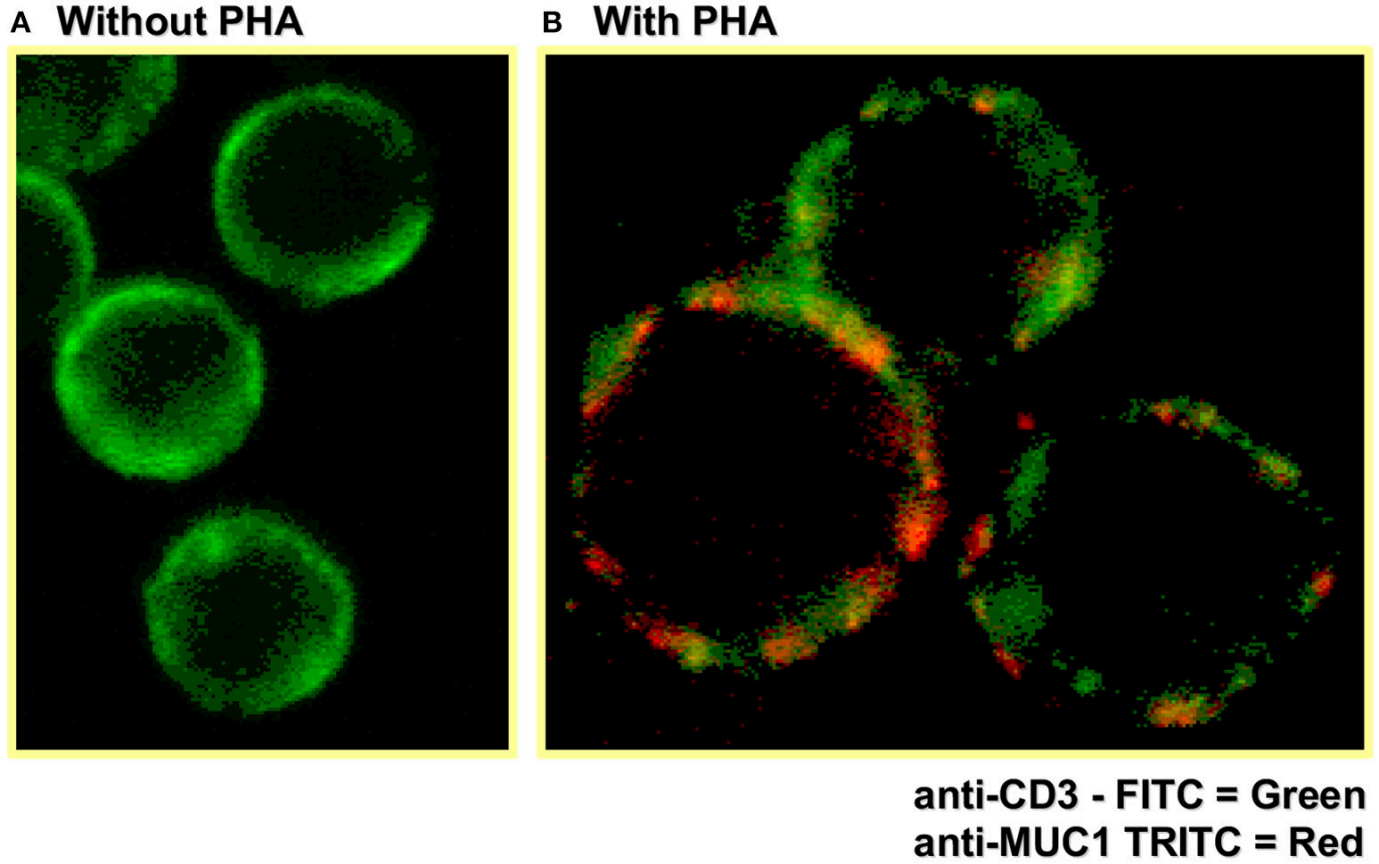
Confocal microscopy shows MUC1 expression on activated human (CD3^+^) T cells. Human T cells were stimulated with PHA for 72 h, followed by staining for CD3 and MUC1 expression using fluorescently labeled antibodies and confocal microscopy.

**Figure 3:**
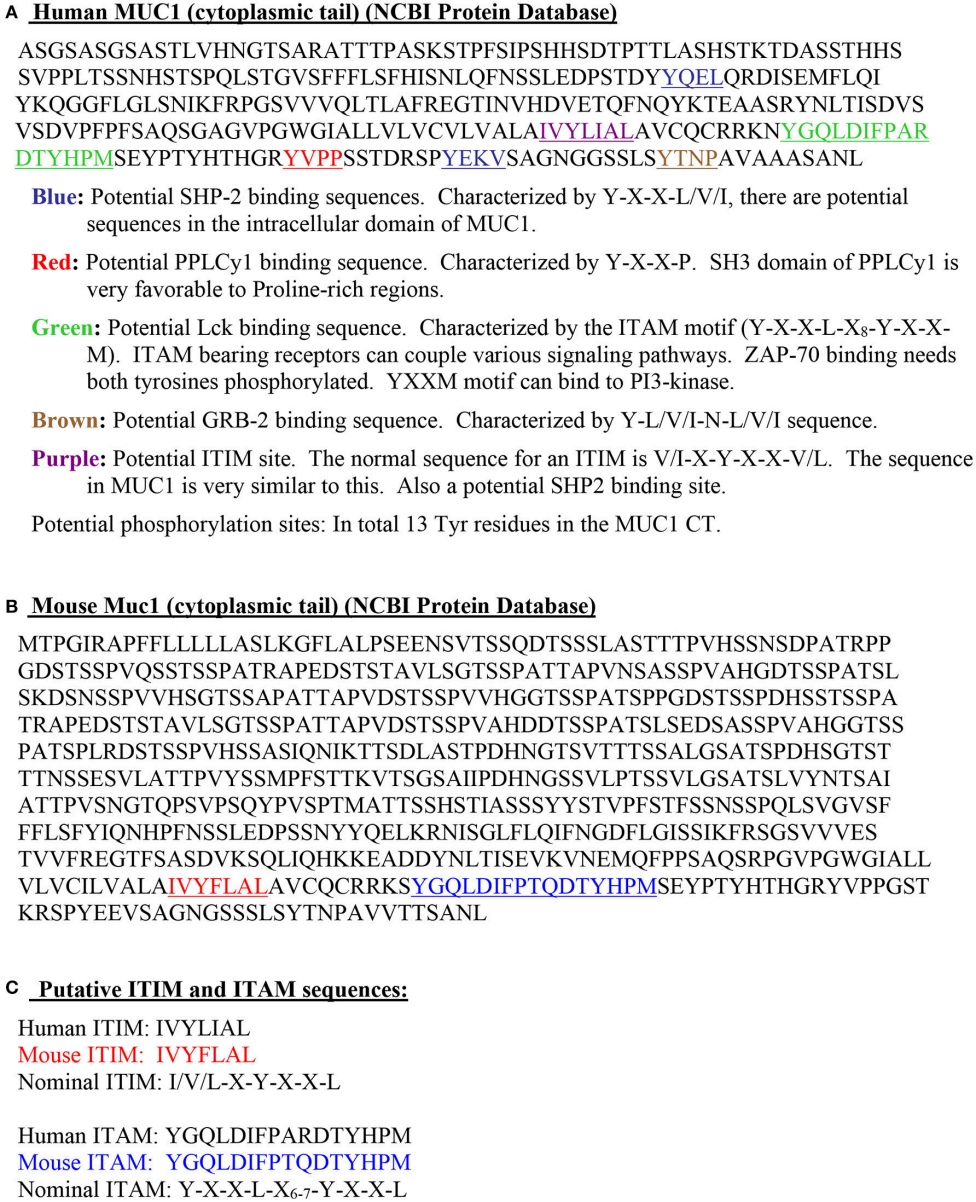
Intracellular cytoplasmic tail of MUC1 demonstrates features supporting its immunoregulatory role. **(A)**. Human MUC1 (cytoplasmic tail; NCBI Protein Database). **(B)**. Mouse Muc1 (cytoplasmic tail; NCBI Protein Database). **(C)**. Putative ITIM and ITAM sequences.

**Figure 4:**
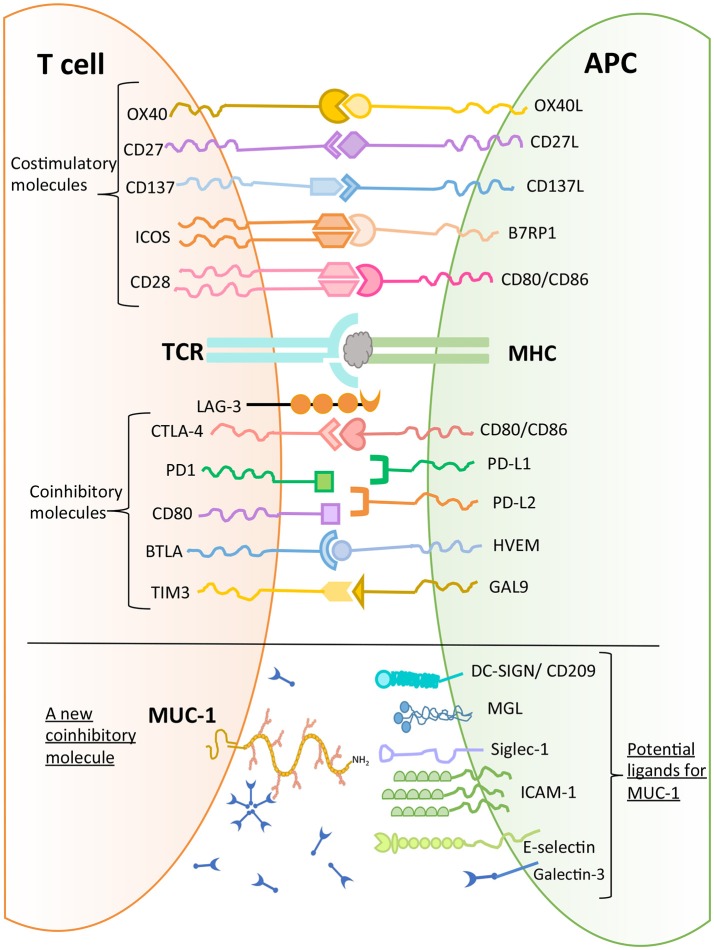
MUC1 as a new coinhibitory molecule on T cells and its putative ligands.

Early flow cytometry analysis of activated T cells, to examine MUC1 expression on CD3^+^ T cells, did not reveal whether TCR complex molecules (e.g., CD3 and MUC1) are co-localized and/or have intracellular cross-talk. Therefore, we also examined the localization of MUC1 in the context of CD3 on the T cell surface by confocal microscopy (Figure 2, unpublished image from 1998). These results showed areas of overlap in anti-CD3 staining and anti-MUC1 staining on the surface of activated T cells, suggesting a possible co-localization of CD3 and MUC1 on the T cell surface. However, co-localization may not necessarily result in molecular interactions between MUC1 and TCR, and must be examined. Further, in this experiment, T cells were stimulated by a T cell mitogen, phytohemagglutinin (PHA); it does not represent localization of MUC1 in the T cell:APC synapse. In our later studies using confocal microscopy, we observed that MUC1 was present on the surface of PHA-activated T cells in a homogenous distribution but relocated and concentrated at a synapse-like juncture after incubating with allogeneic dendritic cells (DCs) for 5 min Agrawal et al., unpublished results. When we examined the cytoplasmic tail of both human and mouse MUC1 molecules, we found the presence of both putative ITAM (Immunoreceptor tyrosine activating motif) and ITIM (Immunoreceptor tyrosine inhibitory motif) like motifs (Figure 3) suggesting that MUC1 could have either a co-inhibitory or a co-stimulatory role. It has been demonstrated that tyrosines on the cytoplasmic tail of MUC1 mucin expressed on activated T cells are phosphorylated, which suggests its plausible role in T cell signaling (43). Interestingly, it has been suggested that both ITIM and ITAM can function to inhibit and/or propagate activation signals such that ITIM can transmit activation and ITAM can inhibit activation (52). Therefore, it still remains to be seen whether and how MUC1 containing these (ITIM and ITAM) motifs participates in regulating T cell responses. It is possible that both of these motifs actually function to strengthen the co-inhibitory role of MUC1 on T cells (Figure 4). The mouse homolog of human MUC1 (Muc1) is only 30% identical with respect to amino acid sequences in the tandem repeat region of the extracellular domain, but is >85% identical in the C-terminal cytoplasmic domain (53), and contains multiple phosphorylation sites along with putative ITIM- and ITAM-like motifs (Figure 3), suggesting an evolutionarily conserved role of the MUC1 cytoplasmic tail across species.

The cytoplasmic tail of MUC1 has been shown to migrate into the nucleus along with transcription factors in tumor cells (54). We therefore hypothesized that MUC1 on T cells may be functioning in a similar manner. Interestingly, upon activation of T cells with anti-CD3 or anti-CD3+anti-MUC1 mAb, the cytoplasmic tail migrated to the nucleus, which also transports AP-1 transcription factors c-Fos and c-Jun (51).

The expression profile of MUC1 on T cells demonstrates an interesting pattern. Among the non-activated T cells, only 3–10% of them express MUC1 by flow cytometry, which gets upregulated upon stimulation with the mitogen PHA (40). However, we have consistently observed that not all of the activated T cells express MUC1 and that inhibition in T cell proliferation mediated by surface MUC1 cross-linking is incomplete except for the mAb BC3 which showed anomalous results of 60% binding but ~99% inhibition (41). This suggests that a subset of T cells is not affected by MUC1 cross-linking either because they don't express MUC1 upon activation or because they are not negatively regulated by MUC1. We examined MUC1 expression on various subsets of non-activated T cells to obtain baseline data on these subsets (51). Upon examining memory and naïve T cell populations, we found that between 10 and 12% of naïve and memory T cells express MUC1 even at the baseline (51). Among the non-activated T cells, a higher percentage of CD8^+^ T cells appear to express MUC1 compared to CD4^+^ T cells (51). Upon activation, however, MUC1 expression is maintained or slightly increased on CD8^+^ T cells except CD8^+^ memory T cells. Contrarily, among the CD4^+^T cells, the percentage of the cell population expressing MUC1 is significantly increased on all naïve, memory, and memory/effector T cells (51). These results clearly show differential expression of MUC1 on CD4^+^ and CD8^+^ T cells, as well as on naïve, memory, and memory/effector T cells. Further studies are now warranted to understand the functional significance of differential expression of MUC1 on various subsets of T cells at different stages.

Our observations with MUC1 parallel those with well-known immune checkpoint molecules PD-1, LAG-3, and TIM-3 that have also been found to be differentially expressed on CD4^+^ and CD8^+^ T cells (55–57). PD-1 expression was reported to be higher on CD4^+^ T cells compared to CD8^+^ T cells (58). In contrast, TIM-3 was shown to be expressed at higher levels on CD8^+^ T cells compared to CD4^+^ T cells (57). CD4^+^ T cells undergoing proliferation that had high expression of PD-1 exhibited lower expression of TIM-3, whereas TIM-3 expressing CD8^+^ T cells had reduced PD-1 expression (57, 58). In addition, expression of these checkpoints was found to differ between cytokine-producing CD4^+^ and CD8^+^ T cells (59).

In another set of experiments, instead of purified T cells, T cells in the presence of APCs were first stimulated with PHA for 72 h to induce MUC1 expression. These T cells were then treated with antibodies against CD3, MUC1, or IgG isotype control and a cross-linking goat anti-mouse antibody. After a three-day incubation, the cells in the MUC1-stimulated group exhibited more proliferation than the anti-CD3 mAb-only group and the IgG isotype control group, with a statistical significance of *p* < 0.01 (51). Anti-MUC1 mAb itself with or without cross-linking did not stimulate T cell proliferation (51). This experiment provided the first evidence that blocking MUC1 by anti-MUC1 mAb leads to removal of the co-inhibitory signals, or alternatively, anti-MUC1 antibody is able to provide co-stimulation to enhance the proliferation normally generated by the anti-CD3 stimulus. Most of the co-stimulatory/coinhibitory molecules of T cells often require CD3 within close proximity due to the sharing of intracellular kinases, phosphatases, and other proteins (60, 61). Using antibody ligated 1 μm latex microspheres to delineate the function of MUC1 co-stimulation, we found that T cell proliferation was enhanced by the anti-CD3 and anti-MUC1 co-ligated beads when compared to the cells treated with separate beads containing the two mAbs (51). The anti-CD3 and anti-MUC1-treated group produced more TNF-α, IFN-γ, and IL-2 into the supernatant compared to the control groups with anti-CD3 alone or anti-CD3 with isotype control and cross-linking antibody (51). It is still not clear whether it is blocking of the inhibitory signals or rather MUC1-mediated co-stimulation. As mentioned earlier, MUC1 can potentially bind to several ligands expressed on APCs. It is possible that instead of providing a co-stimulatory signal, blocking MUC1 by antibodies may act in a signal-independent manner to remove co-inhibition, like anti-CTLA-4 and anti-PD-1 mAbs, by sequestering inhibitory interactions between MUC-1 and its ligands (62–64).

Our observation that CD3 and MUC1 co-inhibition/co-stimulation can modulate T cell responses led us to hypothesize that MUC1 may play a role on regulatory T cells (T_reg_ cells), the primary peripheral regulatory class of lymphocytes (51, 65). We found that approximately 25% of the T_reg_ population (CD4^+^CD25^hi+^FoxP3^+^) expressed MUC1, which after CD3 stimulation, increased to 70–95% (65). Further, we observed that anti-CD3 and anti-MUC1 cross-linking generated a higher percentage of T_regs_ (5–17% of the total gated lymphocyte population) over the control groups (1.5–4%) (65). Interestingly, anti-MUC1 mAb-mediated cross-linking was found to not induce apoptosis in the T cell population (65). T_regs_ are involved in immune homeostasis and maintenance of self-tolerance. In many tumors and chronic infections, they accumulate and represent a major immune inhibitory mechanism. Although transcription factor FoxP3 has been implicated as a Treg marker, it is not unique to T_regs._ Really, there are no cell surface molecules that are unique to T_regs_ (66), but these cells do express high levels of multiple immune-checkpoint molecules, such as CTLA-4, PD-1, TIM-3, LAG-3 etc. (66). Although these checkpoints inhibit effector T cell function, they may serve as effector molecules of T_reg_ cells or promote their differentiation (67–69). In analogy with other checkpoint molecules, cross-linking through anti-MUC1 antibody also significantly expanded putative T_reg_ cells (CD4^+^CD25^+^FoxP3^+^) with the majority of T_regs_ being MUC1^+^ after stimulus, supporting the role of MUC1 as a putative novel regulator of T cells (65). Overall, our studies support our initial hypothesis that MUC1 is a novel putative checkpoint/regulatory molecule, expressed highly on T_regs_ and the blocking of which could lead to enhanced T cell function. It remains to be seen whether MUC1 is highly expressed on T cells in a tumor microenvironment and in conditions of persistent viral/bacterial infection like other T cell coinhibitory molecules (27).

## Experimental evidence supporting the role of MUC1 as an immunoregulatory molecule *in vivo*

Studies from various research groups, using wild-type (WT) and Muc1^−/−^ knockout mice, have shown MUC1 to be associated with an anti-inflammatory/immune-regulatory effect, analogous to other co-inhibitory molecules (70–72).

Muc1 (mouse homolog of human MUC1 is represented as Muc1) knockout mice (Muc1^−/−^) infected with *Pseudomonas aeruginosa* (Pa) showed increased lung injury and the inflammatory mediator cytokines TNF-α and IL-8 in bronchoalveolar lavage fluids compared with control mice (70). The authors concluded that MUC1 may play a crucial role in resolution of inflammation in chronic respiratory infection and that MUC1 dysfunction may likely contribute to chronic inflammatory respiratory disease (70). The mechanism suggested for this effect is as follows: infection with Pa stimulates TLRs, which induce inflammatory cytokines and result in recruitment of macrophages. TNF-α produced during the inflammatory response enhances Muc1 expression in airway epithelial cells, which dampens TLR-mediated inflammation resulting in the return of macrophage numbers to pre-infection levels. In the absence of Muc1 the macrophage influx is maintained, resulting in excessive inflammation. These results indicated an immunoregulatory role of MUC1 expressed on airways and lungs epithelial cells. However, it is possible that besides epithelial Muc1, immunoregulation induced by Muc1 expressed on lymphocytes also amplified these effects. A second study using a Pa infection model in wild-type C57bl/6 and TNFR^−/−^ KO mice showed that upregulation of Muc1 induced by TNF-α during Pa infection may suppress excessive and prolonged inflammatory responses (73).

In another report, it was shown that Muc1^−/−^ mice had increased levels of Th17 cells and IL-17 production and more severe colitis compared to control mice (71). It was suggested that Th17 signaling upregulates MUC1, which in turn functions in a negative feedback pathway to prevent excessive Th17 cell response in inflamed mouse colons. Further, they showed that the TH17 response, which was enhanced in the absence of MUC1, could be abolished if the commensal bacteria were depleted. Therefore, disrupting MUC1-mediated negative feedback pathways may play a role in the development of inflammatory bowel disease (IBD), although gut microbiota participates significantly in this regulation. This study further supported an anti-inflammatory/regulatory role for MUC1.

T cells play an important role in the development of inflammatory responses in multiple sclerosis (MS), an autoimmune disease of the central nervous system. Muc1^−/−^ mice were shown to develop greater experimental autoimmune encephalomyelitis (EAE) symptoms compared to wild-type (WT) mice, with increased numbers of Th1 and Th17 cells infiltrating into the CNS. It was shown that splenocytes from Muc1^−/−^ KO mice had greater amounts of IL-1β, IL-6, and IL-12 but lesser amounts of IL-10 production, compared to wild-type mice (72). It was also shown that splenocytes from Muc1^−/−^ KO mice, which were stimulated with anti-CD3 or anti-CD3 and anti-CD28, expressed higher levels of T-bet, ROR-γ and cytokines IFN-γ and IL-17, compared to WT mice. However, purified T cells isolated from both Muc1^−/−^ and WT mice differentiated similarly to produce IFN-γ and IL-17, whereas DCs isolated from Muc1^−/−^ KO mice produced higher levels of inflammatory cytokines IL-1β, IL-12, and IL-6, and lower levels of IL-10, compared to WT mice (72). These results suggest that lack of Muc1 expression on T cells does not directly influence their ability to produce effector cytokines. However, increased IFN-γ and IL-17 production in Muc1^−/−^ splenocytes could be due to lack of MUC1-mediated regulation on DCs and/or their interaction with T cells, because DCs have also been shown to express Muc1 (72). In this regard, it has been shown that MUC1 interferes with TLR signaling, blocking the interaction with NF-κB and leading to reduction in NF-κB activation and production of inflammatory cytokines (74).

Activated T cells located in the synovial tissue play a significant role in the pathology of rheumatoid arthritis (RA), an autoimmune/inflammatory disease of joints. Aspirates from an acutely inflamed joint of patients with RA contained an increased percentage of T cells expressing MUC1 (48). Similar results were also obtained with aspirates from the joint of a patient with osteoarthritis. These results, for the first time, showed that activated T cells isolated from an ongoing active immune response express MUC1. Further, this study showed that MUC1 is expressed on the leading edge of chemokine-induced T cells, and therefore could have the potential to play a role in T cell migration (48).

These studies support an immuno-regulatory role of MUC1 in infections, inflammation, and autoimmunity (48, 70–72) and further corroborate our seminal findings, establishing MUC1 as a novel T cell coinhibitory molecule with high implications as a novel biomarker and therapeutic target in chronic diseases such as autoimmunity, inflammation and cancer, and possibly infections.

## Expression of MUC1 mucin on other cell types of hematopoietic origin

Interestingly, shortly after our initial finding of MUC1 expression on activated human T cells (40), DCs, B cells, NK cells, bone marrow-derived hematopoietic stem cells, and progenitor cells were also shown to express MUC1 (12). Additionally, bone marrow of Muc1^−/−^ KO mice demonstrated an increased expression of CD11b^+^Gr1^+^ myeloid-derived suppressor cells (MDSCs), which are immune suppressive and support tumor progression. These results suggest that MDSCs not expressing MUC1 expand in the absence of immune regulation brought about by MUC1 (75). Therefore, MUC1 appears to play a role in immune regulation through its expression and function in many different cell types of the immune system, however, intense investigation is required to clearly delineate them.

## Conclusions and future perspectives

Our serendipitous discovery of MUC1 expression on T cells has been not only reproduced by laboratories worldwide but has been expanded to support its role as an important immunoregulatory molecule of T cells, as we initially contemplated. The identification of immunotyrosine-based inhibitory/activation motifs (ITIM/ITAM) sequences in the cytoplasmic tail of MUC1 further supports its function in regulating T cells, in a manner congruent with other coinhibitory molecules such as CTLA-4 and PD-1. Further, as discussed earlier, expression patterns and signaling through MUC1 demonstrated profiles reminiscent of co-inhibitory (checkpoint) molecules of T cells. The *ex vivo* experiments have been corroborated by *in vivo* studies using both human and mouse models of inflammation. In future, there is a clear need to distinctly identify the expression pattern of MUC1, its relation to other co-stimulatory and co-inhibitory molecules, its interaction with various potential ligands and the physiological result of these interactions. This includes determining the potential of carbohydrate side chains of the extracellular domain of MUC1 in interacting with lectins, and the regulation mediated by MUC1 on various subsets of T cells at different points in their lives (naïve, effector, and memory), and in a tumor microenvironment and during chronic infections (exhaustion). These studies would clearly delineate the role of MUC1 in “fine-tuning” T cell responses in immune homeostasis and chronic diseases. Through a more complete understanding of immune regulation through MUC1, we may establish a new biomarker and a therapeutic target to modulate T cell responses in various chronic immunological diseases.

MUC1 antigen has been used as a prominent tumor-associated antigen for the design of various immunotherapeutic strategies. Some of the MUC1-targeted experimental immunotherapeutic approaches are aiming for induction of anti-MUC1 antibodies to clear tumor cells. However, their implications in significantly modulating T cell responses in these patients have not even been conceived yet. There is clearly a need to understand potential effects of generating anti-MUC1 antibody on T cell responses in cancer patients. Manipulating co-inhibition of T cells via anti-MUC1 antibody could be translated as an invaluable tool in counter-acting immune suppression in various cancers.

## Author contributions

BA and NG contributed to the writing of the manuscript. JK is a former graduate student of BA who did significant amount of the experimental work cited in this review article.

### Conflict of interest statement

The authors declare that the research was conducted in the absence of any commercial or financial relationships that could be construed as a potential conflict of interest.
